# PP2C phosphatases—terminators of suicidal thoughts

**DOI:** 10.1038/s41419-024-07269-2

**Published:** 2024-12-19

**Authors:** Lisa Lagorgette, Daria A. Bogdanova, Ekaterina V. Belotserkovskaya, Carmen Garrido, Oleg N. Demidov

**Affiliations:** 1 INSERM, UMR 1231, Laboratoire d’Excellence LipSTIC and « Equipe labellisée par la Ligue Nationale contre le Cancer », University of Burgundy, Dijon, France; 2 University of Burgundy, Faculty of Medicine and Pharmacy, Dijon, France; 3 Division of Immunobiology and Biomedicine, Sirius University of Science and Technology, Sirius University of Science and Technology, Sochi, Russia; 4 Institute of Cytology RAS, St. Petersburg, Russia; 5 Center for Cancer Georges-François Leclerc, Dijon, France

**Keywords:** Cell death, Phosphorylation

## Abstract

Cell death and related signaling pathways are essential during development and in various physiological and pathological conditions. Post-translational modifications such as ubiquitination and phosphorylation play an important role in these signaling pathways. The involvement of kinases - enzymes that catalyze protein phosphorylation - in cell death signaling has been extensively studied. On the other hand, not many studies have been devoted to analyzing the role in cell death of phosphatases, enzymes involved in the removal of phosphorylated residues added to proteins by kinases. Obviously, the two opposite reactions, phosphorylation and dephosphorylation, are equally important in the regulation of protein functions and subsequently in the execution of the cell death program. Here, we have summarized recent work on the involvement of serine-threonine PP2C phosphatases in cell death pathways, senescence and autophagy, focusing in particular on the most studied phosphatase PPM1D (PP2Cδ) as an example of the regulatory role of PP2Cs in cell death. The review should help to draw attention to the importance of PP2C family phosphatases in cell death checkpoints and to discover new targets for drug development.

## Facts

- PP2Cs - is a divergent family of serine-threonine phosphatases involved in the regulation of various physiological and pathological conditions.
- PP2Cs can dephosphorylate the key players of cell death pathways.
- The most studied PP2C phosphatase, PPM1D, is involved in several cell death pathways.

## Open questions

- How do PP2C phosphatases determine the cellular choice of a particular type of cell death?
- Could PP2C phosphatases be replaced by other serine-threonine phosphatases in different signaling pathways?
- What is the role of cellular compartmentalization in the specificity of PP2C phosphatases?

## Introduction into PP2C phosphatases family

**Fig. 1:**
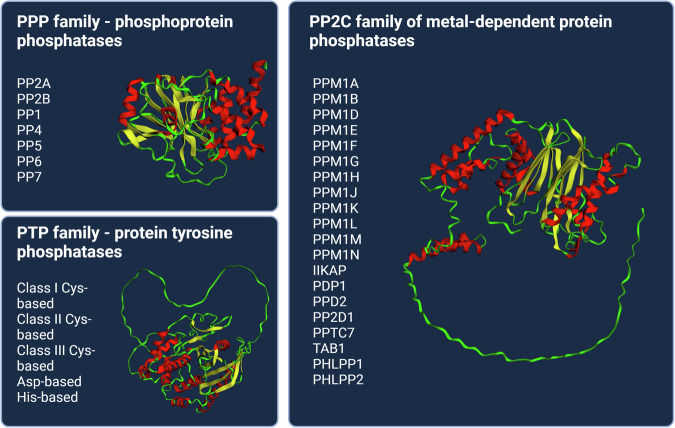
Protein structure and list of the PP2C family members. PP2C family proteins require Mn^2+^ and/or Mg^2+^ for activation. The PP2Cs family is composed of 20 isoforms including PPM1A, PPM1B, PPM1D, PPM1E, PPM1F, PPM1G, PPM1H, PPM1J, PPM1K, PPM1L, PPM1M, PPM1N, ILKAP, PDP1, PDP2, PHLPP1, PHLPP2, PP2D1, PPTC7 et TAB1. The structures of the family member are obtained using Neurosnap Inc. - Computational Biology Platform for Research, Wilmington, DE, 2022 [128] and designed with BioRender.com.

The superfamily of metal-dependent protein phosphatases (PPMs) belongs to the family of 2C-type protein phosphatases (PP2Cs) and includes proteins that dephosphorylate serine and threonine residues [1]. According to different studies, the PP2C members are encoded by 16–18 genes and are represented by 20–22 isoforms [1–3] (Fig. 1). The protein structure of all PP2C is characterized by a catalytic domain bearing certain homology, which is usually located in the N-terminus or the middle of the protein and can be activated by Mn^2+^ and/or Mg^2+^ [4]. The C-terminus of the protein is responsible for localization and substrate specificity [5]. In contrast to PP2A phosphatases PP2Cs do not require targeting by the regulatory subunits, and these enzymes function as monomers [1]. It is worth to mention that different PP2C members can bind to the same substrates, demonstrating overlap in substrate specificity and functions (e.g. p53 binds to PPM1A, PPM1B or PPM1D; ULK1 to PPM1B or PPM1D). Although protein phosphatases of the 2C-type are involved in many signaling cascades, the key functions of these proteins are to control stress-induced responses [6], senescence and cell cycle (growth and death) [1]. PP2Cs can be considered as inhibitors of the stress-induced response [6]. As cell growth regulators, PP2Cs may act as tumor suppressors (ILKAP, PHLPP) [1] or function as activators of oncogenesis (PPM1D) [7].

**Fig. 2:**
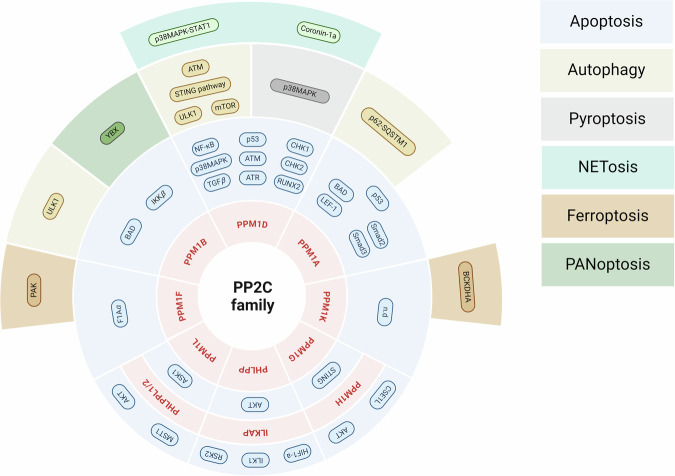
Involvement of the PP2C family members in different types of cell death. The most described PP2C proteins including PPM1D, PPM1A, PPM1K, PPM1F, PHLPP, PPM1L, PPM1G, PPM1B, PPM1H, ILKAP, and PHLPPL1/2 are involved in apoptosis and others cell death pathway. Actually, PPM1D, PPM1A, PPM1K, PPM1F, PHLPP, PPM1L, PPM1G, PPM1B, PPM1H, ILKAP, and PHLPPL1/2 have been reported to regulate apoptosis and PPM1G, PPM1B, PPM1D, PPM1A and PPM1K are involved in autophagy mechanisms. PPM1B play a role in PANaptosis pathway. NETosis and pyroptosis cell death are strictly regulated by PPM1D. Ferroptosis regulation involved PPM1K and PPM1F regulation. All these types of cell death are driven by several signaling pathways and PP2C protein regulate many factors shown in this scheme. N.D. annotation means that there is no data about target of the PP2C family member. Created with BioRender.com.

The PP2C family has been intensively studied since the early 2000s, when it was discovered that these enzymes are involved in the control of apoptosis through various signaling pathways, such as p53 (PPM1A, PPM1D [8, 9]), TGFβ (PPM1A, PPM1D [10, 11]), NF- κB (PPM1B, PPM1D [12, 13]), Bcl-xL (PPM1A, PPM1B [14]), Akt (PHLPP [15]) (Table 1). More recently, PP2Cs have been shown to be involved in other types of programmed cell death, namely autophagy (PPM1A, PPM1B, PPM1D [16–18]); NETosis (PPM1D [19, 20]), ferroptosis (PPM1K [21], PPM1F [22]), PANoptosis (PPM1B [23]) and pyroptosis (PPM1D [24]) (Fig. 2 and Table 1).

**Table 1:** Role of the PP2C family members in programmed cell death and senescence.

As we can see from Fig. 2, the protein phosphatase PPM1D (PP2Cδ) is the most extensively studied in relation to cell death. Using PPM1D as an example of a classical member of the PP2C family, we will describe how phosphatases of this family regulate different forms of cell death and why they have become a promising target in the treatment of various diseases, including cancer therapy. The *PPM1D* gene encodes the wild-type p53-induced phosphatase, PPM1D, which was discovered as a p53 target during the era of intensive studies of p53 post-translational modifications [25]. It soon became clear that phosphorylation positively regulates p53 [26]. In contrast, PPM1D upregulation upon DNA damage creates a negative feedback loop by dephosphorylating p53 and allowing the ubiquitin ligase MDM2 to target p53 for degradation [27]. PPM1D can not only dephosphorylate p53 directly but also target and inactivate many upstream DNA damage response kinases: ATM [28], CHK1 [29], CHK2 [30]. Later, PPM1D was reported to be a phosphatase that targets other important signaling pathways for cell survival, such as mTOR [31], p38MAPK [32, 33], ULK1 [34, 35], NF-κB (RelA) [36, 37], and Coronin-1a [19]. Through its interaction with these targets, PPM1D is involved in cellular processes such as DNA damage response (DDR), cell proliferation, apoptosis, autophagy, NETosis, and senescence. PPM1D may affect key pathways of the senescence that may lead to activation of inhibitors of cyclin-dependent kinases: p16 [23, 32, 38, 39], and p21 [40–43].

## Role of PP2C phosphatases in the regulation of specific types of cell death

### Apoptosis

Considering the importance of the p53-dependent DNA damage response in the initiation of apoptosis and senescence, PPM1D emerged as a negative regulator of these two cellular processes [44]. The human PPM1D coding gene *PPM1D* is located on chromosome 17q23 [24]. This genomic locus is frequently amplified in many human cancers [45, 46]. Alternatively, PPM1D can be stabilized in tumor cells by mutations in the regulatory domain, which, similar to amplification of the gene, affects tumorigenesis [47] and resistance to anticancer therapy [48].

**Fig. 3:**
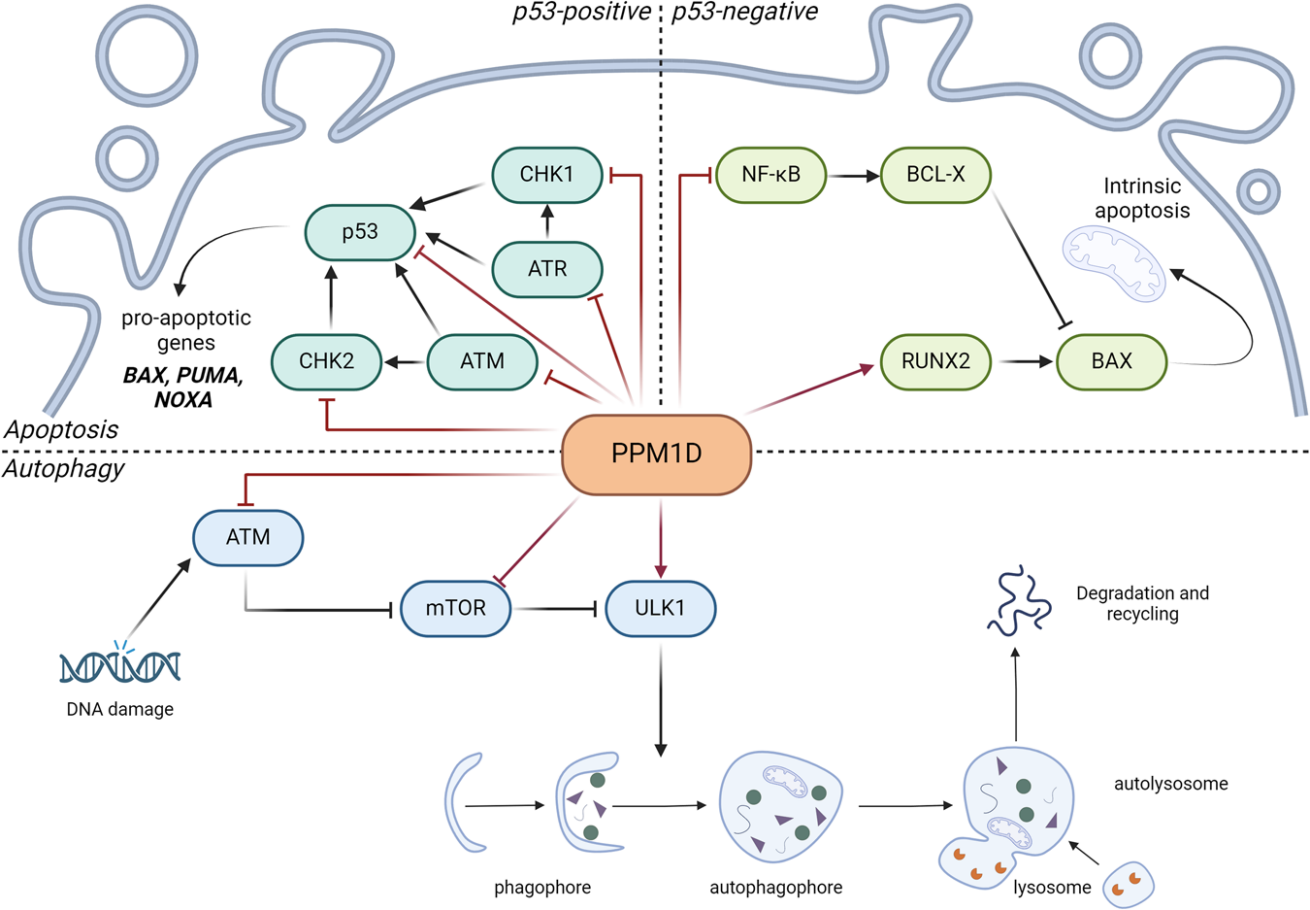
PPM1D-mediated apoptosis and autophagy. PPM1D is a negative regulator of apoptosis by a p53-dependant manner by its direct interaction with ATM, ATR, CHK1, Chk2 and p53. In p53-negative cells, overexpression of *PPM1D* regulates positively apoptosis by its inhibition of NF-κB and activation of RUNX2. Apoptosis activation leads to apoptotic bodies formation responsible for cell death. PPM1D regulated positively autophagy by its inhibitor functions on ATM and mTOR which are negative regulators of autophagy. Otherwise, PPM1D dephosphorylates ULK1 which turns to its activation leading to autophagophore formation responsible for autophagy mechanisms. PPM1D involvement in apoptosis pathway was demonstrated mainly in cancer disease otherwise PPM1D functions in autophagy mechanism were shown in atherosclerosis development. Created with BioRender.com.

P53 is a transcriptional regulator of several pro-apoptotic genes such as *BAX* [49], *PUMA* [50] or *NOXA* [51]. By negatively regulating p53 and its upstream regulators, ATM, CHK2 and others [22, 30, 52], *PPM1D* overexpression protects cells from executing the apoptotic program [53]. Conversely, *PPM1D* deletion significantly shifts the p53 activity threshold toward apoptosis (Fig. 3). Increased p53-dependent apoptosis in Ppm1d-deficient intestinal tumor-initiating stem cells upon DNA damage or oncogenic stress reduced the number of intestinal polyps and prolonged the life of mice with an oncogenic multiple intestinal neoplasia (Min) mutation in the tumor suppressor APC [8]. The phenotype was reversed by deletion of p53 in the tissues of APC (Min)/*Ppm1d*^−/−^ mice to almost the wild-type tumor numbers. A similar phenotype was observed in APC (Min) / *Ppm1d*^−/−^ mice when *p16*, *Chk2* and *Gadd45a* genes were deleted [54].

Interestingly, when the major target of PPM1D, p53, is absent in cells, *PPM1D* deletion does not potentiate programmed cell death [12, 55]. Rather, *PPM1D* overexpression activates *BAX* expression and apoptosis in p53-null osteosarcoma SAOS2 cells by removing inhibitory phosphorylation of the BAX-regulating transcription factor RUNX2 and by reducing anti-apoptotic BCL-XL levels through suppression of the NF-κB pathway [55].

Recently, numerous papers have been published describing PPM1D inhibition as a strategy to potentiate chemotherapy or targeted therapy-induced apoptosis in both solid [8, 28] and hematologic malignancies [48, 56, 57]. It should be stressed, that inhibition of PPM1D alone doesn’t influence cancer cell growth, whereas a combination PPM1D inhibitor with chemotherapeutic drugs or radiation significantly enhances the cytostatic effect of DNA damage agents [56–58].

Except PPM1D, many other PP2C phosphatases could control apoptosis (Table 1 and Fig. 2). Indeed, PPM1A and TAB1 have been described to regulate apoptosis in a p53-dependent manner [9, 59]. The pro-apoptotic protein Bad involved in the pathway could be a substrate for PPM1A and PPM1B in the apoptosis context [14, 60]. AKT has also been described as a substrate of PPM1H and PHLPP in apoptosis [15, 61]. Inhibition of PHLPP increases AKT activity linked to the lower level of apoptosis and B-cell survival in the diabetes context [62].

### Autophagy

Autophagic cell death or autophagy is defined by the NCCD (Nomenclature Committee on Cell Death) as a type of cell death that is dependent on the autophagy machinery and can only be prevented by blocking autophagy [63]. Autophagy is considered an intracellular process aimed at degrading dysfunctional cytoplasmic components thereby recycling nutrients and maintaining cellular homeostasis [64]. This type of cell death starts with the sequestration of detrimental organelles and macromolecules and the formation of a double-membrane autophagosome. It is orchestrated by autophagy-related proteins mainly the unc-51-like kinase 1 (ULK1) complex, consisting of ULK1, ATG13, FIP200/RB1CC1, and ATG10162 [65]. This protein complex, in turn, is controlled by several molecular factors that converge on the mTOR signaling pathway [66]. After the phagophore is formed, it is transported to lysosomes to produce autolysosomes, where the sequestered contents are degraded by lysosomal enzymes [67].

In addition to digesting organelles and proteins, a number of autophagy-mediated processes include oxidative stress, response to cytotoxic agents, cell survival and resistance to infection by pathogens [68].

PPM1D-dependent control of autophagy involves the ATM-mTOR signaling pathway [69, 70]. This has been demonstrated in studies investigating the role of PPM1D in lipid metabolism, cholesterol efflux in macrophages and atherosclerosis [57, 58]. These processes are controlled by the ATM-mTOR cascade [71], which can be modulated by the PPM1D phosphatase through ATM dephosphorylation [52]. Accordingly, in vivo studies demonstrated that *Ppm1d* deletion reduced mTor activation resulting in decreased accumulation of lipid droplets into macrophages. This in turn prevented the formation of foam cells and ultimately atherosclerotic plaques [70].

In addition to ATM kinase regulation, the PPM1D phosphatase has been reported to be involved in basal and genotoxic stress-induced autophagy via ULK-1 dephosphorylation [34, 35] (Fig. 3). As mentioned above, the ULK1 protein complex plays a central role in the induction of autophagy, especially under amino acid starvation. In the presence of amino acids, the ULK1 complex is phosphorylated and inactivated by mTORC1 and AMP-activated protein kinase at different serine/threonine residues [72–74]. Upon amino acid deprivation, ULK1 is dephosphorylated, resulting in activation of the ULK1 kinase and thus autophagy induction [65]. According to two studies [34, 35] the PPM1D phosphatase is responsible for the dephosphorylation of ULK1 at Ser637 and the induction of basal and genotoxic stress-induced autophagy. Using embryonic fibroblasts and primary thymocytes, Torii and co-authors reported that Ppm1d-mediated Ulk1 dephosphorylation was accompanied by Ulk1 puncta formation [35]. In contrast to Torii’s study, Ak’s group explored the breast cancer cell line MCF7, demonstrating *PPM1D* overexpression. They showed that oncogenic PPM1D promoted ULK1-dephosphorylation at Ser637 leading to accumulation of LC3-II, degradation of p62, and formation of LC3-II puncta [34].

Besides ATM-mTOR modulation, PPM1D is involved in regulation of another autophagic molecular mechanism through the STING signaling pathway [17]. It was previously reported that STING was responsible for autophagy [75] and at the same time the STING /TBK1/IRF3 cascade was associated with acute pancreatitis development [76]. In this study, knockdown of *Ppm1d* in rat pancreatic acinar Ficells significantly decreased Sting-Tbk1 signaling, autophagy and acute pancreatitis severity [17]. These data indicate that PPM1D may be involved in autophagy and pancreatitis pathogenesis through the STING-TBK1 signaling pathway.

In summary, PPM1D can modulate “self-eating”, acting as a positive or negative regulator of autophagy depending on the circumstances (Fig. 3).

Similar to PPM1D, the PPM1B phosphatase is also involved in the regulation of amino acid starvation-induced autophagy through dephosphorylation of ULK1 [18]. It has been indicated that under leucine-rich conditions PPM1B is inactive, because this phosphatase is bound to 14-3-3ε protein, but when leucine is scarce, the binding of PPM1B and 14-3-3ε protein is disrupted. Being released, PPM1B becomes active and is able to dephosphorylate ULK1 and initiate autophagy. Interestingly, the involvement of the 14-3-3ε-PPM1B axis in autophagy is associated with a recently identified type of post-translational protein modification such as lysine crotonylation [77]. Leucine starvation has been appeared to stimulate crotonylation of the 14-3-3ε protein, after which the PPM1B - 14-3-3ε interaction is disrupted and autophagy is triggered [18].

Surprisingly, in contrast to the autophagy-activating effect of PPM1D and PPM1B, PPM1A phosphatase downregulates autophagy selectively. A recent study aimed at developing the PPM1A inhibitor has shown that PPM1A phosphatase inhibits *Mycobacterium tuberculosis* selective autophagy in macrophages through dephosphorylation of the autophagy receptor p62/SQSTM-1 [16]

Notably, the *Saccharomyces cerevisiae* orthologs of the human PP2C phosphatases, namely Ptc2 and Ptc3 have also been demonstrated to be involved in the regulation of autophagy. In particular, Ptc2 and Ptc3 phosphatases have been shown to regulate macroautophagy by dephosphorylating the Atg1 kinase complex and promoting autophagy [78].

Taken together, PP2C phosphatases play a significant role in the regulation of autophagy by dephosphorylating various substrates, which mainly results to autophagy promotion.

### NETosis

Neutrophil extracellular traps (NETs) are structures consisting of DNA, histones, proteins from granules, and cytoplasm [79]. Importantly, the main function of NETs is eliminating pathogens, and NET formation is considered a specific type of cell death characteristic of activated neutrophils—NETosis [80].

Previously, it was reported that PPM1D was involved in the development and maturation of neutrophils as a negative regulator via p38MAPK and STAT1 signaling pathways [33]. This study also demonstrated that Ppm1d participated in the antibacterial defense, migration of neutrophils and inflammatory processes [33]. In line with these findings, the negative effect of Ppm1d on neutrophil migration and antimicrobial defense was confirmed in *Ppm1d*^−/−^ mice under sepsis conditions [81]. Hereafter, PPM1D was revealed to be involved in nuclear reorganization during NETosis [20]. Most intriguingly, a recent study found that PPM1D could inhibit NET formation during infection through the calcium pathway [19]. Investigating of the mechanism of the Ppm1d negative effect it was revealed the Coronin-1a was a substrate of Ppm1d for direct dephosphorylation. Upregulation of phosphorylated Coronin-1a in neutrophils in which Ppm1d was deleted promoted phospholipase C activation and the calcium signaling pathway [19]. Taken together, Ppm1d is a negative regulator of NETosis and can be considered as a potential target for antibacterial therapy.

### Ferroptosis

Ferroptosis is a specific form of iron and lipid peroxidation-dependent cell death [82]. Induction of ferroptosis is widely discussed in the context of therapeutic strategies for the treatment of cancer, including lung cancer [83]. Several modulators of ferroptosis are regulated by the proto-oncogene transcriptional coactivator YAP and stimulate ferroptosis [82]. PPM1F has been shown to dephosphorylate and negatively regulate PAK (p21^Cdc42/Rac^-activated kinase) [84]. In lung cancer, an axis has been shown in which increased transcription of PPM1F inhibits PAK, which contributes to the inhibition of ferroptosis by decreasing YAP activity [85]. Another PP2C family phosphatase, PPM1K, have been shown to be associated with ferroptosis in the brain. Specifically, decreased expression of PPM1K in neurons in vitro can lead to increased levels of ferroptosis in neurons [21]. Although the role of PPM1D in ferroptosis has not yet been studied, PPM1D participation in an iron-dependent form of cell death can be hypothesized based on the involvement of PPM1D targets such as p53 [86] and ATM [87].

### Pyroptosis

Pyroptosis represents a further type of programmed cell death. The principal effector proteins involved in the pyroptosis cascade are the caspase-1-activating proteins called gasdermins. These proteins result in the formation of pores and the disruption of cell membrane integrity, which ultimately leads to cell death [88]. In the recent article [89], the role of the phosphatase PPM1D as a negative regulator of the pyroptosis pathway was examined [89]. In a model of LPS-induced kidney injury, it was demonstrated that the inhibition of Ppm1d resulted in an increase in cell death [89]. The p38MAPK signaling pathway plays a role in regulating pyroptosis [89]. Inhibition of PPM1D activity may contribute to renal tubule damage by stimulating phosphorylation of p38MAPK [89]. In this instance, there is an observable increase in the levels of key proteins associated with the NLRP3 pathway, including cleaved caspase 1, GSDMD-N, and IL-1β [89]. Moreover, it has been previously shown that overexpression of *PPM1D* can effectively mitigate LPS-induced inflammatory factor release and protect the integrity of the blood-brain barrier from LPS-induced damage in an in vitro model [90].

### PANoptosis

PANoptosis represents a novel form of cell death that exhibits characteristics of apoptosis, necroptosis, and pyroptosis [91]. The role of PANoptosis as a response to antitumor therapy is discussed, and the importance of studying the proteins involved in the regulation of this process is emphasized [92]. A study demonstrated that the YBX1 protein inhibits PANoptosis and may enhance resistance to oxaliplatin-based chemotherapy [93]. It is noteworthy that this process is regulated by PPM1B [93]. PPM1B dephosphorylates YBX1, thereby inhibiting its activity [93]. This results in a reduction in YBX1 levels, which in turn affects the efficacy of the cellular response to oxaliplatin [93]. As previously described, PP2C family phosphatases regulate numerous cell death processes, suggesting a potential for identifying their leading regulatory role in PANoptosis in the future.

### Senescence

PPM1D is interesting in the senescence process because it is an inhibitor of key proteins involved in the DNA damage response (DDR). Among these proteins, the p53-p21 axis plays a specific role in the induction of the senescent phenotype [94] and, as shown in several articles, the state of senescence can also be mediated by the p38MAPK-p16 axis [32, 39, 95–97]. Senescence is characterized by permanent cell cycle arrest caused by increased expression of inhibitors of the cyclin-dependent kinase proteins p16 and p21 [98]. At the same time, the senescent cells remain metabolically active and secrete a wide range of pro-inflammatory cytokines and chemokines, termed the senescence-associated secretory phenotype (SASP) [98]. SASP exerts multiple negative effects on both oncogenesis and normal aging [99]. The removal of the negative effects of senescent cells is a field of intensive research [100]. PPM1D may be expected to be an important target and regulator of these processes.

#### p21 axis

One of the most significant contributions to this field was the work focused on *Ppm1d*^−/−^ mouse embryonic fibroblasts (MEFs) [40]. *Ppm1d*^−/−^ MEFs were shown to undergo premature senescence at physiological O_2_ concentrations [40]. The onset of senescence was dependent on the functional activation of p53 [40]. Interestingly, *Ppm1d*^−/−^ MEFs demonstrated increased levels of H2AX phosphorylation without increased levels of reactive oxygen species (ROS) or DNA base damage, which were present in wild-type MEFs [40]. These data demonstrate that Ppm1d prevents the induction of cellular senescence at physiological oxygen levels by attenuating DDR signaling in response to endogenous double-strand breaks (DSBs) that occur during DNA replication [40]. This hypothesis has latter on been confirmed in other cell types. For example, Ppm1d deficiency leads to upregulation of H2AX and cellular senescence in the hippocampus [101]. Another article highlights that the cellular stress response to elevated ROS during long-term monolayer culture of chondrocytes is enhanced by decreased *Ppm1d* expression, and it may trigger the onset of cellular senescence [102].

The absence of PPM1D leads to increased DDR signaling activity as a result of endogenous DSBs detected during the S phase. Interestingly, in several human tumor cell lines, Ppm1d protein levels progressively increased from G1 to G2 phases and then decreased during mitosis, revealing the importance of Ppm1d in regulating the nature of the cellular response to DDR [103]. Similar data have been obtained in a mouse model of progeria mice [104]. In Zmpste24-deficient mice (*Zmpste24*^*−/*^^−^ mice), a Hutchinson-Gilford model of progeria syndrome, increased miR-29 expression was found both in progeroid *Zmpste24*^*−/*^^−^ mice and during normal aging of wild-type mice [104]. The authors concluded that this transcriptional activation of miR-29, which suppresses the Ppm1d, is triggered in response to DNA damage and occurs in a p53-dependent manner [104].

The state of cellular senescence plays an important role in tumor control and is often mediated by the p53-p21 axis. Therefore, most studies focus on the role of these proteins, overlooking PPM1D, which is the major inhibitor of p53. PPM1D inhibitor has been proposed as therapeutic approaches for some cancers [105]. The use of PPM1D inhibitor results in the restoration of p53 activity. However, recovery of p53 does not always induce apoptosis, for instance in soft tissue sarcomas and hepatocellular carcinoma, the initiation of the p53 cascade induces senescence followed by immune-mediated tumor elimination [106, 107]. Similarly, in a model of lung adenocarcinoma, p53 restoration initiates a senescence response followed by immune-mediated clearance, but only in advanced tumor lesions [108]. Further, pharmacological inhibition of Ppm1d increases p21 protein levels, one of the major markers of senescence [41–43]. Perhaps the combined use of PPM1D inhibitor and senolytics, which selectively eliminate senescent cells, may be an effective strategy for different tumor types [100]. Investigating PPM1D inhibitors in the context of tumor cell senescence may open up new treatment options for patients, and such strategies may have fewer side effects. This may be of particular interest in the context of oncohematologic diseases, where PPM1D may be overexpressed in clonal hematopoiesis [109]. Despite recent advances in the treatment of oncohematological diseases in the elderly, the median survival rate remains low and the risk of tumor relapse remains high [110]. It is increasingly thought that sensitized cancer cells are the cause of these relapse statistics, which persist in bone marrow niches and can re-enter the cell cycle afterwards due to a large number of accumulated mutations and genome instability [57, 111, 112]. This re-entry into the cell cycle, following the acquisition of a senescent phenotype, which should be an irreversible state of cell cycle arrest, is a characteristic feature of tumor cells of various origins [113, 114]. It has been demonstrated that this process may be regulated by Ppm1d [115]. *Ppm1d* expression is reduced during the acquisition of the senescent phenotype. This reduction is necessary for the establishment of a permanent cell cycle arrest. Conversely, the reintroduction of *Ppm1d* expression results in the resumption of the cell cycle [115]. The aforementioned findings were obtained in a model of Therapy-Induced Senescence (TIS), which is particularly characteristic of tumor cells. TIS is a phenomenon that occurs during cancer treatment, through the use of radiotherapy, immunotherapy, and chemotherapy [114]. A reduction in *PPM1D* expression and an increase in p53 activity have been demonstrated to be essential for the induction of TIS in human cancer cells by chemotherapeutic drugs [115].

One of the adverse consequences of the accumulation of senescent cells within a tumor is the secretion of SASP factors [116, 117]. Roberson et al. observed that prematurely senescent cells, which overexpress *Ppm1d*, develop amplified SASP [113]. The data indicate that p53 is a significant regulator of SASP, with cells lacking p53 secreting higher levels of several SASP components [118]. Previously, Leonardi’s laboratory also suggested that, consistent with the ability of PPM1D to inhibit some important functions of p53, forced expression of *PPM1D* led to increased expression of a subset of cytokines and chemokines in prematurely senescent cells, but the exact role of PPM1D and the mechanisms of this modulation require further study [115]. These data are supported by an earlier review, which illustrated that PPM1D activity decreased with age, likely due to a decline in p53 function. This increases the activity of the p38MAPK and NF-κB pathways, leading to premature cellular senescence and chronic inflammation in some tissues [37]. However, the relationship between PPM1D and SASP has not been directly investigated.

Other phosphatases have also been demonstrated to act as regulators of the p53-p21 pathway. However, in this context, the PPM1D phosphatase remains the most extensively studied, exhibiting a prominent role as a negative regulator of p53. For example, it has been demonstrated that TRIM22-mediated degradation of PHLPP2 results in the activation of AKT-p53-p21 signaling, which ultimately induces cellular senescence in hepatocellular carcinoma cells [119]. In turn, PPM1G controls p21 expression through the PI3K/AKT pathway, which also affects senescence processes [120, 121]. PPM1K has been demonstrated to regulate glycolysis and hematopoietic stem cell quiescence via the pathway MEIS1/p21 [122]. However, direct evidence for its involvement in the regulation of senescence processes remains inconclusive.

#### p16 axis

A number of studies have shown that inhibition of p38MAPK activity under various stress conditions prevents senescence or growth arrest by blocking p16 expression [32, 39, 95–97]. P16 is an inhibitor of cyclin-dependent kinases and a major marker of cellular senescence [32]. P38 mitogen-activated protein kinases (p38MAPK) are a class of threonine mitogen-activated protein kinases [123]. p38MAPK regulate a variety of cellular processes, including cell cycle regulation, cell death induction, differentiation, and cellular senescence [123].

One of the earliest publications in 2004 elucidated that *Ppm1d*^−/−^ MEFs were resistant to transformation in the presence of different pairs of complementary oncogenes, including *Ras, Myc, E1a* and *Erbb2* [32]. Bulavin et al concluded that inactivation or depletion of *Ppm1d* phosphatase and subsequent activation of p38MAPK suppressed carcinogenesis by modulating the *p16* tumor suppressor locus [32].

The same results have also been obtained using human cancer cell lines. In human breast cancer tissues, PPM1D expression levels were shown to be inversely correlated with p38MAPK activity and low p16 levels [39]. It has also been found that in breast cancer cell lines with reduced expression of PPM1D, there is an increase in p38MAPK activity and an increase in HBP1 protein levels, thereby inducing senescence [124]. These data confirm the role of PPM1D in regulating p38MAPK signaling in senescence and subsequent tumor suppression [124]. However, this study was focused on HBP1, not p16, in p38MAPK-induced premature senescence [124]. The authors proposed that HBP1 did not regulate replicative senescence as has been shown in human fibroblasts [124]. Another investigation clarified that introducing PPM1D into normal human mesenchymal stem cells (MSCs) bypasses senescence and extends the lifespan of the cells [23]. The bypassing of such growth arrest and replicative senescence may be due to PPM1D-induced reduction of *p16* expression, as introduction of PPM1D into MSCs significantly reduced p38MAPK activation and *p16* expression that occur in senescent MSCs [23]. It was demonstrated that p21, one of the downstream effectors of the p53 senescence pathway, was not induced in senescent MSCs, implying that senescence is mediated by the p38MAPK -p16 axis [23].

The use of mouse models altered for p38MAPK and/or Ppm1d has yielded new insights into the mechanisms of p16-dependent regulation of cell proliferation with age [38]. It has been shown that during the aging process, a reduction in Ppm1d protein level leads to an increase in p38MAPK activation [38]. Pancreatic β-cells have been demonstrated to suppress *p16* transcription through the action of Bmi1, which is present in the *p16* locus [38]. However, in senescent cells, p38MAPK signals MK2/MK3 kinases to phosphorylate Bmi1, which in turn activates *p16* and *p19* transcription [38]. The expression of cell cycle inhibitors results in a reduction in the proliferation and regeneration of certain self-renewing cell types [38]. Additionally, it has been reported in mouse models that young (4–5 months old) *Ppm1d*^−/−^ mice and old (22–24 months old) wild-type mice exhibited a considerable elevation in p38MAPK-dependent signaling [38]. Furthermore, the *Ppm1d* overexpressing mouse model demonstrated a reduction in p38MAPK phosphorylation and a decline in *p16* and *p19* mRNA levels, which resulted in pancreatic islet proliferation [38]. Consequently, PPM1D may be considered as a significant physiological regulator of p38MAPK signaling during senescence.

Furthermore, PPM1B has been demonstrated to influence the aging process, with its levels observed to decline progressively with age. A reduction in PPM1B levels in human fibroblast cultures has been demonstrated to result in the development of a stable senescence phenotype [125]. It is postulated that PPM1B serves as a regulator of both p38MAPK-dependent and independent pathways during replicative cell senescence [125].

**Fig. 4:**
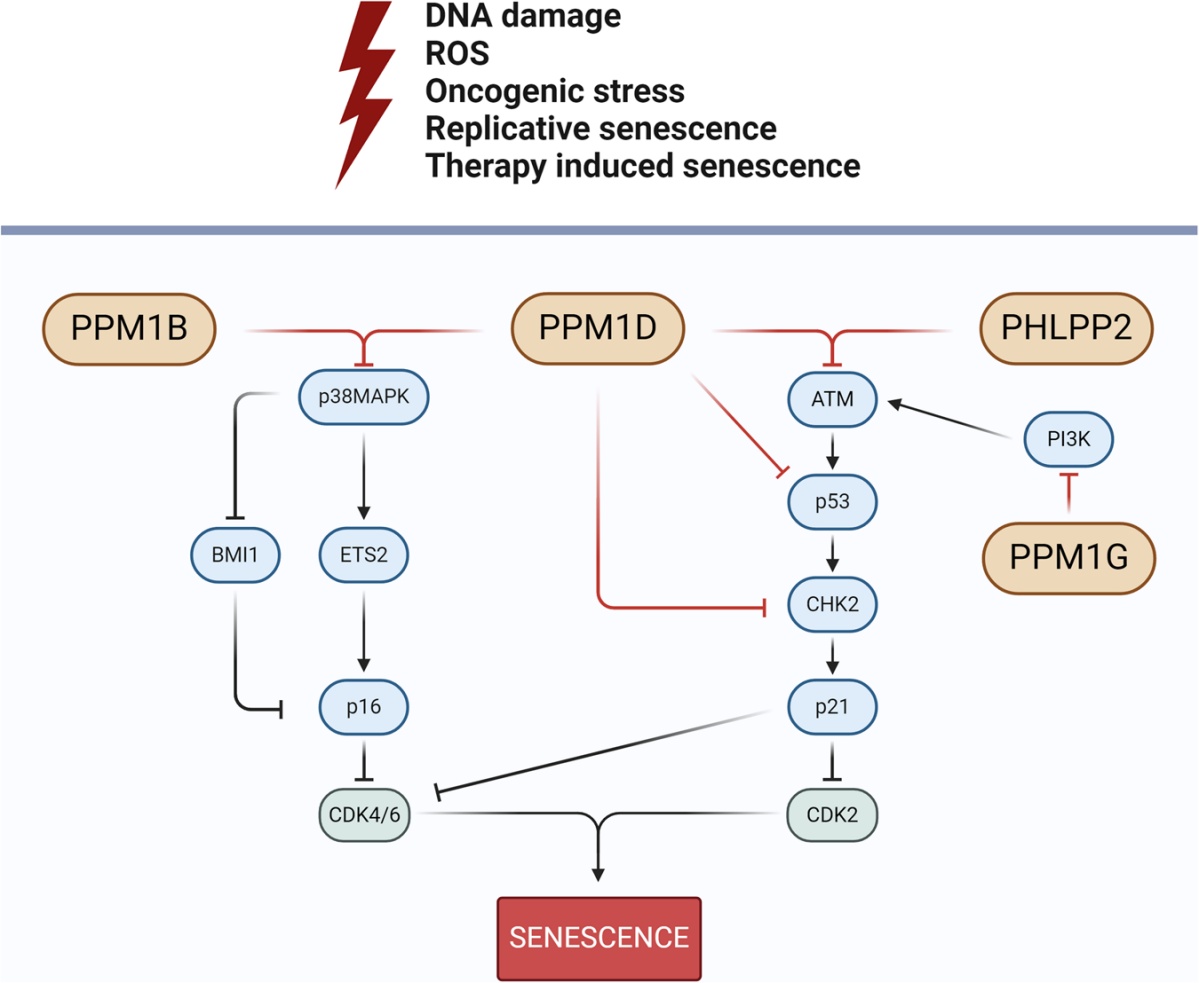
Schematic of the two main molecular cascades of senescence regulated by PP2C family members. In p53-p21 axis, PPM1D regulates senescence mainly by its direct negative interaction with ATM, p53 and CHK2 turning to p21 reduction which decreases senescence induction. The p53-p21 axis can also be regulated by PHLPP2 by inhibiting AKT and by PPM1G by inhibiting PI3K, which activates AKT-p53-p21. In a p38MAPK -p16 axis, PPM1D and PPM1B act as a negative regulator through their interaction with p38MAPK leading to p16 reduction which turns to senescence decrease. Created with BioRender.com.

Figure 4 displays two major cascades that are involved in the process of senescence and are regulated by PP2C. A comprehensive investigation of the function of all PP2C family phosphatases in cellular senescence and carcinogenesis will facilitate the identification of more effective targets and alternative cancer treatment strategies. The predominant role of PPM1D as the most studied PP2C family phosphatase and a major negative regulator of p53 is further emphasized by an article published in 2024 [126]. The authors delineate the role of PPM1D in tumor cell transformation by regulating senescence and cell death [126]. We suggest that the potential use of PPM1D inhibitors to induce senescence in cancer cells with preserved p53 and/or p38MAPK is of considerable interest. The resulting senescent cells could be eliminated with senolytics, which may serve as a new strategy for the treatment of cancer. In this case, PPM1D inhibitors may prove effective not only in cancer patients with wild-type p53, as previously hypothesized, but also in those with p53 deficiency. A novel mechanism underlying the therapeutic efficacy of PPM1D inhibitors has been identified in a model of p53-deficient non-small cell lung cancer. It is hypothesized that these inhibitors may exert effects beyond p53 and be effective regardless of p53 status [127].

## Conclusion

As described above, the accumulating evidence points to PPM1D as a negative regulator of different types of cell death. It is becoming apparent that other members of the PP2C family may act in a similar manner to PPM1D and may replace PPM1D as negative regulators of pro-death molecules in signaling pathways. Therefore, inhibition of PP2C phosphatases is considered a potential therapeutic strategy in various diseases, for example, to potentiate chemotherapy or targeted therapy-induced apoptosis in both solid and hematological malignancies. We predict that the development of new PP2Cs specific inhibitors in the near future will make this potential strategy a reality and make the treatment of various diseases more efficient.
